# Orthogeriatric treatment reduces potential inappropriate medication in older trauma patients: a retrospective, dual-center study comparing conventional trauma care and co-managed treatment

**DOI:** 10.1186/s40001-019-0362-0

**Published:** 2019-01-22

**Authors:** Johannes Gleich, Daniel Pfeufer, Christian Zeckey, Wolfgang Böcker, Markus Gosch, Christian Kammerlander, Carl Neuerburg

**Affiliations:** 1 Department of General, Trauma and Reconstructive Surgery, University Hospital, LMU Munich, Marchioninistr. 15, 81377 Munich, Germany; 2 Department of Medicine 2/Geriatrics, Paracelsus Private Medical University, General Hospital Nuremberg, Nuremberg, Germany

**Keywords:** Integrated care, Hip fracture, Humeral fracture, Pharmacotherapy, Frailty

## Abstract

**Background:**

Multimorbidity and polypharmacy are common challenges in the treatment of older trauma patients. Therefore, various integrated care models were developed over the last few years, merging the expertise of geriatricians and trauma surgeons. The aim of this study was to evaluate, if the number of prescriptions of potentially inappropriate medication (PIM) could be reduced in these patients by an interdisciplinary co-managed concept compared to conventional trauma care.

**Methods:**

We conducted a retrospective, dual-center cohort study, including all patients aged 70 years and older admitted with a fracture of the hip or the proximal humerus within the study period. Patients were treated in the universities department of trauma surgery with two different hospital sites, one with conventional trauma care (CTC) and the other one with a certified orthogeriatric trauma unit (OGC). Based on the STOPP/START criteria by O´Mahony et al., PIMs were defined, which should be avoided in (ortho)geriatric patients. Medical records of each patient were analyzed at discharge. Besides patients basic information, all prescribed drugs, changes in the medication plan and who carried out these changes were collected. For statistical analysis based on the data quality and distribution, the *t* test, Mann–Whitney U test and the Chi-square test were used.

**Results:**

A total of 95 patients were included, 73 of them females, with an average age of 82.59 years (SD ± 6.96). Mean length of hospital stay was 12.98 at CTC and 13.36 days at OGC (*p* = 0.536). Among conventional care (41 patients), prescription of one or more PIMs was found in 85.4% of the patients, whereas at the orthogeriatric ward (54 patients) only in 22.2% (*p* < 0.001). Besides that, changes in medication were made for 48.1% of the patients during their stay on the orthogeriatric ward.

**Conclusions:**

Our findings show that an integrated care concept can reduce the number of prescriptions of PIMs significantly and potentially avoids adverse drug reactions and additional burdens in older trauma patients.

## Background

The number of older trauma patients is expected to increase in the next years, with hip and humeral fractures as two of the most frequent fractures in these patients [1]. For hip fractures, a global increase up to 6.3 million/year until 2050 is stated [2]. Besides the fracture itself, frailty, multimorbidity and resulting polypharmacy (up to 44% of patients at an age of 65 years or older) bring additional difficulties for patients as well as for trauma surgeons and their team [3, 4]. Especially in the early phase of injured older patients and those undergoing surgery, vulnerability is very high and the use of potential inappropriate medication (PIM) could further worsen this situation. Poor outcomes with mortality rates up to 25–30% in the first year after hip fracture, nearly 10% one year after humeral fracture and a decrease in the abilities for activities of daily living are common [5–7]. Gosch et al. [8] could show the impact of medications on the long-term outcome of older hip fracture patients; thus, prescription of one PIM leads to an increased relative risk of mortality by 28%. Regarding to Laboni et al. [9] with 51% of their older patients receiving at least one PIM, prescription of PIMs was common after hip fracture surgery and was associated with a longer time to achieve full recovery. These results are also seen in other surgical specialties like general-, gynecological- und urogenital surgery with prescription of PIMs in 19–23% of older patients undergoing surgery [10]. Although these difficulties are well known, there is still disagreement about the best way of treatment for older trauma patients. Four different care models are described, firstly a trauma ward with consultation of a geriatrician on demand, secondly trauma wards with regular consultation by a geriatrician, thirdly a geriatric ward with regular consultation by a trauma surgeon and the fourth, most intensive model of an orthogeriatric ward with interdisciplinary management, which represents the highest level of co-management [11, 12]. By now, this co-managed model, where trauma surgeons and geriatricians work together on a specifically designed ward with a specialized team of nurses, physiotherapists and many others, remains the most effective approach, which showed improvements in mortality rates, mobility and independency [13–15].

Therefore, the present study was performed based on the hypothesis that an interdisciplinary treatment reduces PIMs in older adult trauma patients.

## Methods

We conducted a retrospective, dual-center cohort study at a level I trauma center. The clinic has two different sites, one with conventional trauma care (CTC) (49 beds) and the other with a certified orthogeriatric unit (OTC) (44 beds), where the patients are treated in an interdisciplinary manner such as the complex ward model described by Pioli [11].

Thus, permanent integration of the geriatrician into the trauma team was granted, including daily interdisciplinary ward rounds (except on weekends, as there is no geriatrician at service and consultation of specialists of internal medicine is provided on demand) of each orthogeriatric patient and considering all elements of orthogeriatric care (according to Lisk et al.) [16]. Integration starts with the inpatient admission and ends at discharge. Medication was checked by the geriatrician at admission to hospital (medication plan brought by patient, interview with patient/relatives/general practitioner), at the daily interdisciplinary meeting of the orthogeriatric team and at discharge. PIMs were defined, which should be avoided in older trauma patients, based on the STOPP/START criteria presented by O´Mahony et al. and the PRISCUS list by Holt et al. (Table 1) [17–19].

**Table 1:** Most relevant PIMs in orthogeriatric setting

Drug	Reason
NSAID (Ibuprofen, Diclofenac, Aspirin)	Increased risk for: gastrointestinal ulcer, myocardial infarction (MI), stroke, hypertensive crisis, impaired renal function
Benzodiazepines	Increased risk for sedation, delirium, falls
Tricyclic antidepressants at preexisting dementia	Increased risk for delirium, falls, urinary retention, cardiac arrythmia
Opiates (long acting) at preexisting dementia	Cognitive worsening
Antimuscarinic drugs at preexisting dementia	Cognitive worsening
Neuroleptics (long acting) at preexisting Parkinson´s disease	Cognitive worsening
SSRI-type antidepressants at hyponatremia	Cognitive worsening, increased risk of falls, negative effect on bone metabolism
Antibiotics Gyrase inhibitors Gentamicin	Increased risk for: QT extension, seizure, dizziness, confusion, tendon rupture Renal failure, ototoxicity
Antihistamines (especially H1)	Increased risk for: constipation, dizziness, cognitive impairment
Statins (except at preexisting MI, coronary heart disease, stroke)	Muscle weakness, increased risk for rhabdomyolysis
Urologicals/incontinence medication (except indispensable)	Increased risk for: cognitive worsening, falls
Glucocorticoids (except indispensable)	Confusion, negative effect on bone metabolism
Digitalis (except indispensable)	Cardiac arrythmia
Diuretics (except indispensable, e.g., at renal insufficiency)	Increased risk for: dizziness, dehydration, confusion, electrolyte imbalance
Specific antihypertensive drugs (clonidine, reserpine, propranolol, hydralazine)	Increased risk for: cognitive impairment, depression, orthostatic hypotension, sedation

At the conventional trauma ward, treatment was managed by trauma surgeons with no specific geriatric expertise, following the current guidelines and other departments were consulted in case of need.

All consecutive patients aged 70 years and older, who were admitted due to a fracture of the hip or the humerus from 07/2016 until 09/2016, were included. There were no other in- or exclusion criteria, to give a realistic view of the patient collective. The study was approved and registered by the local ethics committee of Munich university (Reg. No. 234-16); no informed consent had to be obtained because of retrospective data collection. Demographic data and important values for medication (glomerular filtration rate (GFR), creatinine level, vitamin D level, Charlson Comorbidity Index, ASA score, time to surgery) were collected from the hospital information system and checked for comparability of the two groups. After discharge, medical records of each patient were analyzed, especially information about basic medication at admission, changes in the medication plan while their hospital stay including the person, who carried out these changes (geriatrician or trauma surgeon) and medication at discharge were collected.

Primary outcome parameters were the number of prescription of PIMs, changes in the medication plan and start of an osteoporosis treatment as secondary outcome.

All data were merged in a special built data sheet (Excel 2011, Version 14.0 for Mac OS X, Microsoft Cooperation, Redmond).

Data were reported as mean ± standard deviation, categorical data as absolute frequency with a percentage distribution. Chi-square test was used for statistical analysis of categorical variables; *t* test and Mann–Whitney test were used depending on data distribution. A *p* value < 0.05 was stated as statistically significant. IBM SPSS Statistics for Macintosh, Version 24 (IBM Corp. Released 2016. Amonk, NY) was used for the statistical analysis.

## Results

A total of 95 patients were included, 77% (73) of them were females. Table 2 shows baseline characteristics of the patients. No statistically significant differences were found in between the two groups except gender (Table 2).

**Table 2:** Baseline characteristics

	CTC (*n* = 41)	OGC (*n* = 54)	*p* value
Age (years), mean (SD)	81.54 (± 6.91)	83.39 (± 6.959)	0.201
Gender (female)	87.8% (*n* = 36)	68.5% (*n* = 37)	0.027
ASA score			0.406
1–2	29.3% (*n* = 12)	22.2% (*n* = 12)	
3	68.3% (*n* = 28)	74.1% (*n* = 40)	
4–5	2.4% (*n* = 1)	3.7% (*n* = 2)	
Charlson comorbidity index, mean (SD)	2.51 (± 1.989)	2.09 (± 1.733)	0.281
Length of stay (days), mean (SD)	12.98 (± 4.43)	13.36 (± 5.516)	0.536
Proximal humerus	11.00 (3.661)	10.00 (6.453)	0.656
Femoral	13.70 (4.519)	14.67 (4.817)	0.392
Type of fracture			0.440
Proximal humerus	26.8% (*n* = 11)	22.2% (*n* = 12)	
Femoral			
Trochanteric	29.3% (*n* = 12)	29.6% (*n* = 16)	
Femoral neck	41.5% (*n* = 17)	37.0% (*n* = 20)	
Periprosthetic	2.4% (*n* = 1)	11.1% (*n* = 6)	
Type of treatment			0.218
Femoral			
Osteosynthesis	50.0% (*n* = 15)	50.0% (*n* = 21)	
Prosthesis	50.0% (*n* = 15)	50.0% (*n* = 21)	
Humeral			
Osteosynthesis	54.5% (*n* = 6)	91.7% (*n* = 11)	
Prosthesis	45.5% (*n* = 5)	8.3% (*n* = 1)	
Time to surgery			
Femoral			0.134
< 24 h	93.3% (*n* = 28)	81.0% (*n* = 34)	
24 h	6.7% (*n* = 2)	19.0% (*n* = 8)	
Humeral			0.901
< 24 h	27.3% (*n* = 3)	25.0% (*n* = 3)	
> 24 h	72.7% (*n* = 8)	75.0% (*n* = 9)	
GFR (ml/min)	66.93 (± 19.865)	69.93 (± 21.474)	0.496
Creatinine (mg/dl)	0.945 (± 0.357)	1.11 (± 0.453)	0.056
Vit. D			0.213
< 20 ng/ml	69.2%(*n* = 18)	84.4% (*n* = 27)	
> 20 ng/ml	30.8%(*n* = 8)	15.6%(*n* = 5)	
Not stated	*n* = 15	*n* = 22	
Discharged to			
Femoral			0.777
Own home	13.3% (*n* = 4)	14.3% (*n* = 6)	
Nursing home	10.0% (*n* = 3)	4.8% (*n* = 2)	
Rehabilitation unit	63.3% (*n* = 19)	71.4% (*n* = 30)	
Short-term nursing	13.3% (*n* = 4)	9.5% (*n* = 4)	
Humeral			0.169
Own home	72.7% (*n* = 8)	58.3% (*n* = 7)	
Nursing home	0% (*n* = 0)	16.7% (*n* = 2)	
Rehabilitation unit	9.1% (*n* = 1)	25.0% (*n* = 3)	
Short-term nursing	18.2% (*n* = 2)	0% (*n* = 0)	

**Fig. 1:**
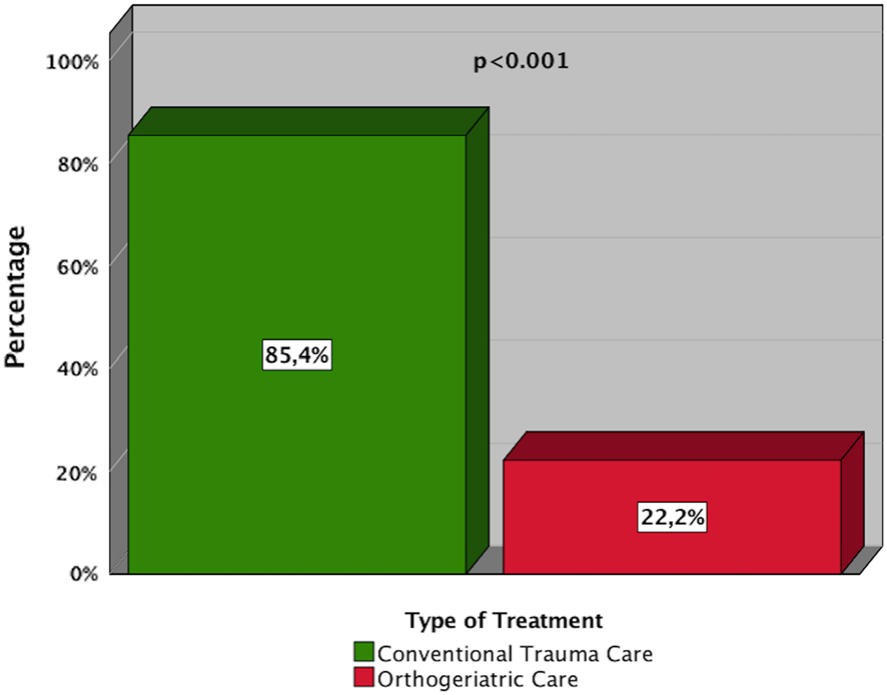
Use of PIMs: Illustration of the use of PIMs in either OGC- or CTC-treated patients

**Fig. 2:**
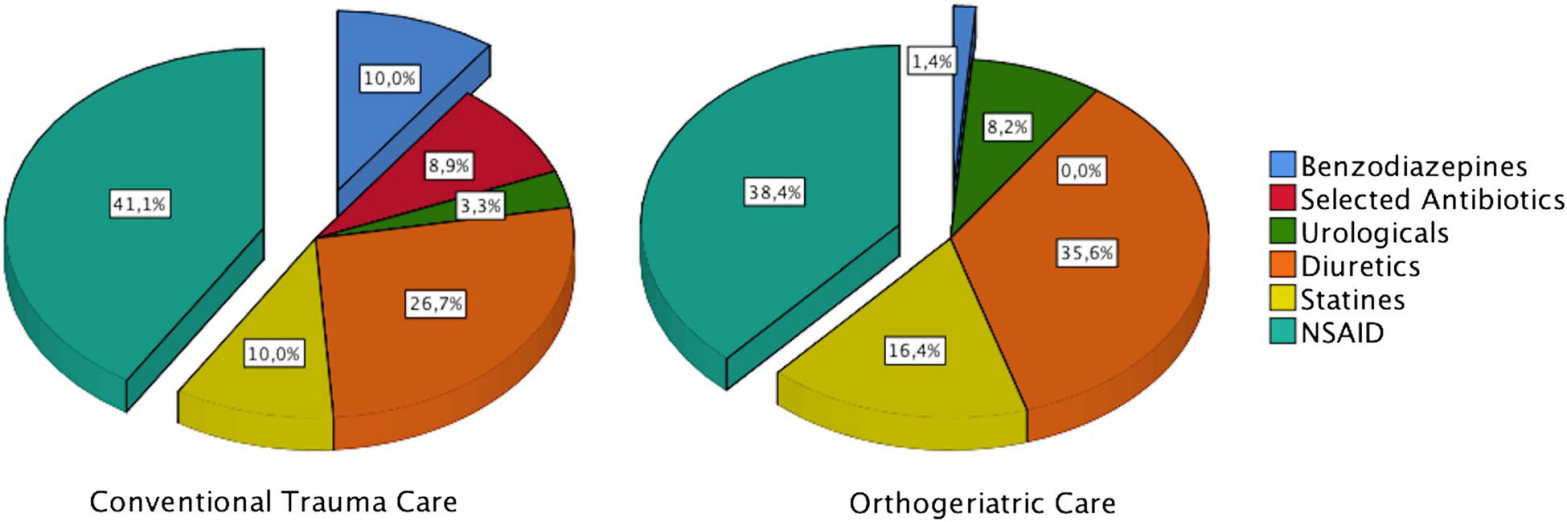
Distribution of PIMs: see Table 1 for detailed description of each group of drugs/their adverse effects

Prescription of a PIM was registered in 85.4% (35 of 41 patients) at the conventional trauma ward and in 22.2% (12 of 54 patients) at the orthogeriatric ward (*p* < 0.001) (Fig. 1). Frequency of distribution of the most used drugs is shown in Fig. 2.

While on the conventional trauma ward no adjustment of medication during hospital stay was observed, 48.1% (26 of 54 patients) of the patients on the orthogeriatric ward received an adjustment by the geriatrician.

Preexisting osteoporosis medication was registered in 19.5% (*n* = 8) of the patients with conventional and 16.7% (*n* = 9) with integrated care (*p* = 0.72). During their stay in hospital, diagnostic evaluation of osteoporosis status (blood values and bone density measurement) was performed in 41.5% (*n* = 17) with conventional and 42.6% (*n* = 23) of the patients with orthogeriatric treatment.

No significant difference regarding cognitive impairment at admission was observed (CTC: 17.1%, *n* = 7; OGC: 14.8%, *n* = 8; *p* = 0.765).

## Discussion

Orthogeriatric patients frequently present with polypharmacy (up to 44% as mentioned above) and are prone to secondary complications arising from potential inappropriate drug prescription. Certain drugs are particularly hazardous in these patients, such as benzodiazepines which can potentially cause confusion, delirium and further cognitive disorders. The present study aimed to evaluate differences in the prescription habits in older trauma patients undergoing an interdisciplinary co-managed care compared to patients being treated by conventional trauma care, without any input by a geriatrician, in a level-one department of trauma surgery with two different hospital sites.

An additional input to conduct the present study was the case of an 86-year-old female who was treated on our orthogeriatric ward. The patient was admitted with a proximal femoral fracture, various comorbidities and, therefore, an intake of numerous drugs. Given two different concentrations and product names of two existing lithium dosages, the preexisting medication was mistakenly adopted and transferred by the staff. During her stay in hospital, increasing creatinine blood levels were observed, which were discussed on the daily interdisciplinary ward round with our geriatrician. After controlling medication and additional blood values, a lithium intoxication was found, which was previously not detected by the surgeons and medication was subsequently paused for the in-hospital period; renal function recovered within a few days.

Regarding the number of prescriptions of PIMs, a significant difference between orthogeriatric and conventional trauma care was observed in the present study. Patients treated on an orthogeriatric ward received a more cautious prescription of medication as those primarily treated by surgeons. This might be attributed to various factors. At first, careful re-evaluation of patients medical records by the geriatrician at admission to the orthogeriatric ward, where the preexisting medication plan, which might already include inappropriate prescriptions, is checked. This procedure is also carried out by the staff on the conventional trauma ward, but they may not have the specific geriatric expertise in interaction of various pharmaceuticals and their effect on older patients. Secondly, daily interdisciplinary ward rounds and discussions of each patient at OGC help to manage upcoming difficulties like postoperative delirium and find the right medication in conjunction with the current medication plan. This is represented by registered adjustments to the medication by the geriatrician in almost half of the patients at OGC. Thus, significantly more changes during hospital stay were observed on the OGC compared to those treated with CTC. There may also be a learning process for trauma surgeons on an OGC. Consistent input of the geriatrician improves the surgeons understanding for the risk of adverse drug reactions and how to avoid them as shown previously [20].

Regarding fracture treatment, no difference between the two hospital sites was observed. The data suggest that there was no relevant influence of the geriatrician to the surgical treatment plan in the early stage. This might be explained by the time when the geriatrician is involved in the interdisciplinary team. At OGC, this is usually short after surgery and only few situations might result in an earlier involvement. In frail patients with more complex fracture patterns, geriatricians might be involved earlier to give additional input for choosing a specific surgical procedure or decline surgery in individual cases.

Regarding secondary fracture prevention, both hospital sides provide a fracture liaison service (FLS), which coordinates diagnostics, treatment initiation and continuity for an underlying osteoporosis. Patients who suffer a fragility fracture and present with special risk factors for an osteoporosis are included and guideline adapted treatment is recommended [21]. Therefore, the detected rate of osteoporosis assessments is comparable in both hospital sites.

The importance of secondary fracture prevention is also highlighted by a study of Ryg et al. [22] who could show recurrent fractures following primary hip fracture. Besides the FLS approach to improve bone quality and prevent fragility fractures, this includes adjustment of medication to decrease induced symptoms like dizziness, which is often followed by falls and removal of fall risk-increasing drugs. Negative side effects of the most frequently used PIMs according to the STOPP/START criteria are as follows. Antibiotics like gyrase inhibitors and diuretics increase the risk of dizziness [23–25], although a use was observed in less than 10% of the patients treated with CTC. Benzodiazepines and opioids are frequently used in the pre- and postoperative stage and of undisputed benefit for anxiety and pain relief, yet they should be stopped as soon as possible because of their large potential to induce falls and therefore their impact on mortality [26–28]. Numerous studies on NSAIDs show their negative impact on cardiovascular system and the increased risk for major bleedings, despite intake of proton-pump inhibitors [29, 30]. They appeared to be the major proportion of PIMs detected in both CTC- and OGC-treated patients, which also might be associated with their beneficial effect, i.e., for the prophylaxis of periarticular ossification in hip fracture patients.

Although the surgical treatment remained comparable, which is a strength of the study for the interpretation of the input arising from the geriatrician, it contains some limitations. Given the retrospective study design, there might have been inconsistent documentation of prescribed and discontinued drugs, which could distort our findings. Also, only the detection of PIMs was evaluated, while further investigation if the hazardous effect of the individual prescription occured, was refrained. On the other side, bias of the prescription habits of the staff was excluded, while a prospective study design might be accompanied by specific attention of the staff, which could affect analysis. Another aspect of interest, which could not be reliably obtained, is patients’ medication at admission. Evaluation of the preexisting medication plans of each study center revealed that there was inconsistent documentation, which is partly attributed to the patients’ cognition or medication plans were forgotten back home by either the patients themselves or the ambulance service. This would have given a better perspective on the impact of the geriatrician on PIM from admission to discharge and should be considered for following prospective studies on that topic.

It would also be of interest for further studies to assess the impact of geriatricians in older trauma patients presenting with a fracture usually requiring a shorter stay in hospital, like a distal radius fracture. In these cases, the impact of the geriatrician might be limited. Although the interdisciplinary co-managed concept might not be feasible for every hospital due to personal limitations of the staff on trauma wards, further education of trauma surgeons with regards to selected drug prescriptions is recommended, given the findings of the present study and increasing numbers of elderly trauma patients.

## Conclusion

Integration of a geriatrician into a trauma ward has shown significant benefits for older trauma patients. Despite already-known positive impact on mortality, preservation of independency, activities of daily living and mobility, reduced prescriptions of PIMs, improved selection of medication and an increased awareness for orthogeriatric patients were observed.

Especially, more fall risk-increasing drugs like benzodiazepines and medication causing dizziness were recorded in patients treated with CTC compared to prescriptions at OGC. Consequently, cautious selection of medication in older trauma patients is likely to prevent adverse drug reactions and reduce hazardous complications and may improve the long-term outcome. Regarding secondary fracture prevention, this is a key factor besides reliable osteoporosis evaluation and management to reduce fragility fractures.
